# A Data-Driven Response Virtual Sensor Technique with Partial Vibration Measurements Using Convolutional Neural Network

**DOI:** 10.3390/s17122888

**Published:** 2017-12-12

**Authors:** Shan-Bin Sun, Yuan-Yuan He, Si-Da Zhou, Zhen-Jiang Yue

**Affiliations:** 1 School of Aerospace Engineering, Beijing Institute of Technology, Zhongguancun South Street 5, Beijing 100081, China; sunshanbin114@sina.com (S.-B.S.); appleyuanyuan@bit.edu.cn (Y.-Y.H.); mountain_yue@bit.edu.cn (Z.-J.Y.); 2 Key Laboratory of Dynamics and Control of Flight Vehicle, Ministry of Education, Beijing 100081, China; 3 Key Laboratory of Autonomous Navigation and Control for Deep Space Exploration, Ministry of Industry and Information Technology, Beijing 100081, China

**Keywords:** virtual sensor, convolutional neural network, partial vibration measurements, response transmissibility

## Abstract

Measurement of dynamic responses plays an important role in structural health monitoring, damage detection and other fields of research. However, in aerospace engineering, the physical sensors are limited in the operational conditions of spacecraft, due to the severe environment in outer space. This paper proposes a virtual sensor model with partial vibration measurements using a convolutional neural network. The transmissibility function is employed as prior knowledge. A four-layer neural network with two convolutional layers, one fully connected layer, and an output layer is proposed as the predicting model. Numerical examples of two different structural dynamic systems demonstrate the performance of the proposed approach. The excellence of the novel technique is further indicated using a simply supported beam experiment comparing to a modal-model-based virtual sensor, which uses modal parameters, such as mode shapes, for estimating the responses of the faulty sensors. The results show that the presented data-driven response virtual sensor technique can predict structural response with high accuracy.

## 1. Introduction

In engineering applications, measurements of vibration responses are essential for structural health monitoring, damage detection, and active control. With current technology, the number of sensors is often limited and the locations may also be inaccessible for instrumentation, especially in the context of aerospace engineering. Under operational conditions, the surface of a spacecraft is unavailabe to install sensors because of the severe environment and the aerodynamic constraints. For instance, in outer space, the temperature on the Sun-facing surface is rather high, while the temperature on the dark side is really low. Therefore, the surface temperatures may be beyond the range of the physical sensors. Besides, the wiring of the sensors in spacecraft is really difficult. Thus, in these cases, the number and locations of the sensors are limited, and to acquire as much information from the limited sensors is of much importance.

To obtain the desired responses using limited physical measurements, virtual sensing techniques have developed rapidly in the last decades. Virtual sensors were originally introduced in process industry to predict hard-to-measure variables, which may be caused by a lack of sensors or the high cost of sensors, using easy-to-measure variables [1,2]. Over the decades, it became a hot research subject and has been proven to be a powerful tool in many industrial processes. There are basically two types of virtual sensors, model-based virtual sensors and data-driven virtual sensors [3]. Model-driven virtual sensors are based on equations describing the relations between the input and output variables. Several model-based virtual sensors were introduced for sensing responses in an engineering structure [4,5,6]. In these model-based virtual sensing, finite element models are needed. However, for large and complex engineering structures, the generation and validation of accurate finite element models requires great effort.

Data-driven virtual sensors train black-box models based on the historical real-life measurement data and use them in industrial processes. Compared to model-based virtual sensors, data-driven virtual sensors require little prior mechanical knowledge but rather a large amount of real-life measured data to train the generated model. In the field of aerospace engineering, spacecraft have to undergo various structural dynamic experiments in the laboratory before launching. Besides, the environment in the laboratory is much milder than in the outer space, making it possible to install sensors at the locations of interest to measure responses that are not available under real operational conditions. Thus, it is possible to train the virtual sensor model with that abundant measurement data. In the area of structural dynamics, Kullaa introduced a data-driven virtual sensor for validating and reconstructing faulty sensors [7]. The method can successfully detect, identify and reconstruct faulty sensors in a sensor network.

In the last decades, machine learning methods have drawn considerable attention. Both kernel methods, e.g., support vector machine [8], and artificial neural network [9,10] have proven to be efficient nonlinear approximators and become popular research areas. Data-driven virtual sensors using various machine learning algorithms have also achieved good performance in a variety of areas [11,12,13,14,15].

Transmissibility is related to revealing the relation between responses from different locations. However, studies have proven that the transmissibility functions differ in different load cases. Since the load cases in operational conditions are always unknown and changing, transmissibility cannot to be directly used for predicting vibration responses using real-time responses from other locations. Thus, this paper attempts to combine machine learning algorithms with transmissibility functions as a prior knowledge and presents a data-driven virtual sensor technique with partial vibration measurements using convolutional neural network.

This article is organized as follows. Section 2 reviews the transmissibility functions, which is employed as the prior knowledge for building the virtual sensor model. In Section 3, the convolutional neural network (CNN) is briefly introduced as the nonlinear estimator, and the architecture of the virtual sensor model is proposed. Then, Section 4 presents two different numerical examples to demonstrate the performance of the novel algorithm. In Section 5, an experimental example is conducted, and a modal-model-based virtual sensor is employed to indicate the good performance of the method. Section 6 briefly discusses the selection of activation functions and hyperparameters of the proposed model. Finally, in Section 7, the conclusions are drawn and future work is discussed.

## 2. Transmissibility Functions and Its Time-Domain Representation

Transmissibility reveals the relation between two response spectra in one structural system. In this section, the concept and property of multivariable transmissibility are summarized. Then, the time-domain representation of transmissibility is given as the main inspiration of the proposed virtual sensor model.

In Section 2.1, the definition and formulation of the multivariable transmissibility are reviewed. In Section 2.2, transmissibility is extended to time-domain using a time series autoregressive model with exogenous input (ARX).

### 2.1. Multivariable Transmissibility

Transmissibility is defined as the ratio between two response spectra [16,17,18]. In Laplace domain, the transmissibility function $X_{i}\left(s\right)$ $T_{ij}\left(s\right)$ is defined as the ratio between two output responses and $X_{j}\left(s\right)$:

(1) $$T_{ij}\left(s\right)=X_{i}\left(s\right)/X_{j}\left(s\right)$$

In a multiple-input-multiple-output (MIMO) system, the relation between the responses X(s) and excitation forces U(s) is given by a transfer function matrix as shown in Equation (2).

(2) X(s)=H(s)U(s)

By separating the responses into reference responses $X_{r}\left(s\right)$ and non-reference responses $X_{q}\left(s\right)$, Equation (2) can be transformed into the following equation:

(3) $$\left[\begin{array}{c} X_{r}\left(s\right)\\ X_{q}\left(s\right) \end{array}\right]=\left[\begin{array}{c} H_{r}\left(s\right)\\ H_{q}\left(s\right) \end{array}\right]U\left(s\right)$$

And the transmissibility functions of an MIMO system are defined as

(4) $$X_{q}\left(s\right)=T\left(s\right)X_{r}\left(s\right)$$

where T(s) indicates the multivariable transmissibility functions [19]. Combining Equations (3) and (4), yields:

(5) $$T\left(s\right)=H_{q}\left(s\right){\left[H_{r}\left(s\right)\right]}^{-1}$$

where ${\left[H_{r}\left(s\right)\right]}^{-1}$ is assumed as either the inverse or pseudo-inverse of $H_{r}\left(s\right)$ as the case may be.

Thus, the transmissibility functions of a MIMO system are obtained. One of the advantages of transmissibility functions is that it is proven to be independent of the input spectrum [20]. Therefore, it indicates that the non-reference responses can be obtained using reference responses ignoring the type of excitation once transmissibility functions are determined.

### 2.2. Time-Domain Representation of Transmissibility

In engineering conditions, vibration sensors measure time domain data. Thus, it is hard to utilize frequency domain transmissibility functions for response prediction of a system. Hence, a time-domain representation of the transmissibility functions is required.

The autoregressive model with exogenous inputs (ARX) has been a successful tool for time series analysis and system identification. An ARX model, with $n_{A}$ auto-regressive orders and $n_{X}$ exogenous input orders, is represented by the following expression,

(6) $$\sum_{i=0}^{n_{A}}A_{i}z^{i}\cdot x\left(t\right)=\sum_{j=0}^{n_{X}}B_{j}z^{j}\cdot u\left(t\right)+e\left(t\right)$$

where $A_{i}$ and $B_{j}$ are parameter matrices, x(t) and u(t) are structure responses and exogenous inputs respectively, z is the backshift operator which can be presented as $z^{i}\cdot x\left(t\right)≜x\left(t-i\right)$, e(t) is the residual error of the model, and t presents the *t*-th discrete time.

In one certain load case, replace x(t) and u(t) with non-reference responses $x_{q}\left(t\right)$ and reference responses $x_{r}\left(t\right)$ respectively, consider e(t) as equation errors, the relation between the reference responses and non-reference responses can be obtained by the following equation,

(7) $$\sum_{i=0}^{n_{A}}{\tilde{A}}_{i}x_{q}\left(t-i\right)=\sum_{j=0}^{n_{X}}{\tilde{B}}_{j}x_{r}\left(t-j\right)$$

where ${\tilde{A}}_{i}$ and ${\tilde{B}}_{j}$ are the estimated values of $A_{i}$ and $B_{j}$ in Equation (6) respectively. Thus the non-reference responses can be predicted as

(8) $$x_{q}\left(t\right)=-\sum_{i=1}^{n_{A}}{\left[{\tilde{A}}_{0}\right]}^{-1}{\tilde{A}}_{i}x_{q}\left(t-i\right)+\sum_{j=0}^{n_{X}}{\left[{\tilde{A}}_{0}\right]}^{-1}{\tilde{B}}_{j}x_{r}\left(t-j\right)$$

where ${\tilde{A}}_{0}$ is the value of ${\tilde{A}}_{i}$ in Equation (7) when i=0.

Equation (8) cannot be used for predicting responses under operational conditions since the transmissibility functions are different in various load cases. However, it can be used as a guidance of structure of a nonlinear approximator, e.g., neural networks, to generate a virtual sensor for operational conditions.

However, one major problem of the transmissibility functions is that they are not identical with varying locations of external excitations. Under operational conditions, the load case of a structure always varies with time and environments, thus the changing of transmissibility functions is unavoidable. Therefore, it is not feasible to predict responses using the transmissibility functions directly.

## 3. Virtual Sensor Model Using CNN

Inspired by the time-domain representation of the transmissibility functions introduced in Section 2.2, a virtual sensor model using convolutional neural network is introduced. In Section 3.1, basic ideas of convolutional network are reviewed. Then, Section 3.2 gives the architecture of the virtual sensor.

### 3.1. Convolutional Neural Network

Neural networks have seen great development in the area of artificial intelligence during the last several decades to deal with machine learning problems like classification and regression. Convolutional networks are a kind of neural networks specialized for processing data with a known, grid-like topology [21].

Convolutional neural networks usually consist of a number of stages, include a convolutional layer, a nonlinear layer and a feature pooling layer. These stages are used for extracting features from the input, thus the output are called feature maps. After several stages, the architecture is followed by other machine learning methods for solving regression problems or classification problems.

The basic idea of convolution layer is simply using convolution instead of general matrix multiplication in the layers. The so-called convolution in CNN is actually the cross-correlation operation. Giving a two-dimensional input I and a two-dimensional kernel K, the cross-correlation output S is defined as:

(9) $$S_{ij}=\sum_{m}\sum_{n}I_{\left(i+m\right)\left(j+n\right)}K_{mn}$$

**Figure 1:**
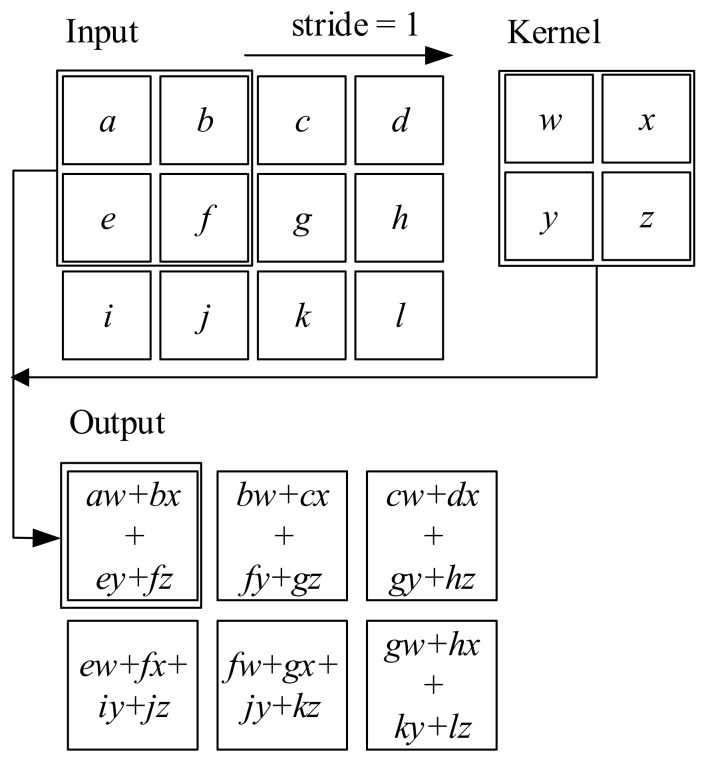
Convolution in a convolutional layer.

In a convolutional layer, a 3D array input with $n_{1}$ 2D feature maps of size $n_{2}\times n_{3}$ is scanned by a trainable kernel (filter) with a certain step size (usually called stride), as shown in Figure 1. A kernel is a 3D array, consists of $n_{1}$ 2D kernels of size $l_{i}\times l_{2}$. In a convolutional layer, $m_{1}$ kernels are used. The output of the convolutional layer is also a 3D array with $m_{1}$ 2D feature maps of size $m_{2}\times m_{3}$. And the output y can be obtained by

(10) $$y_{j}=b_{j}+\sum_{i=1}^{n_{1}}k_{ij}*x_{i}$$

where $y_{j}$ and $x_{i}$ denote the *j*-th output feature map and *i*-th input feature map, respectively, $k_{ij}$ is *i*-th 2D kernel in the *j*-th 3D kernel, $b_{j}$ is a trainable bias parameter and * is the 2D convolutional operator.

Usually, a convolution output is always followed by a nonlinear activation function. In this paper, tanh function is employed. The tanh function is defined as

(11) $$\tanh\left(x\right)=\frac{e^{x}-e^{-x}}{e^{x}+e^{-x}}$$

A pooling layer is used for extracting important features in each feature map to reduce the size of the feature maps when dealing with large image data. However, in this paper, the data are discrete time series vibration measurements, besides the size of input data is rather small. Thus, there is no need to add pooling layers in the convolutional network.

### 3.2. Architecture of the Virtual Sensor Model

In the virtual sensor model, raw input data are time-history response series from several reference sensors. To meet the requirement of the convolutional neural network, the input of the reference response data can be reconstructed into two-dimensional $n_{t}\times s$ matrices, where $n_{t}$ is the number of total discrete time for the data, s is the number of reference sensors. Thus, the input data contains information in both time dimension and sensor dimension.

**Figure 2:**
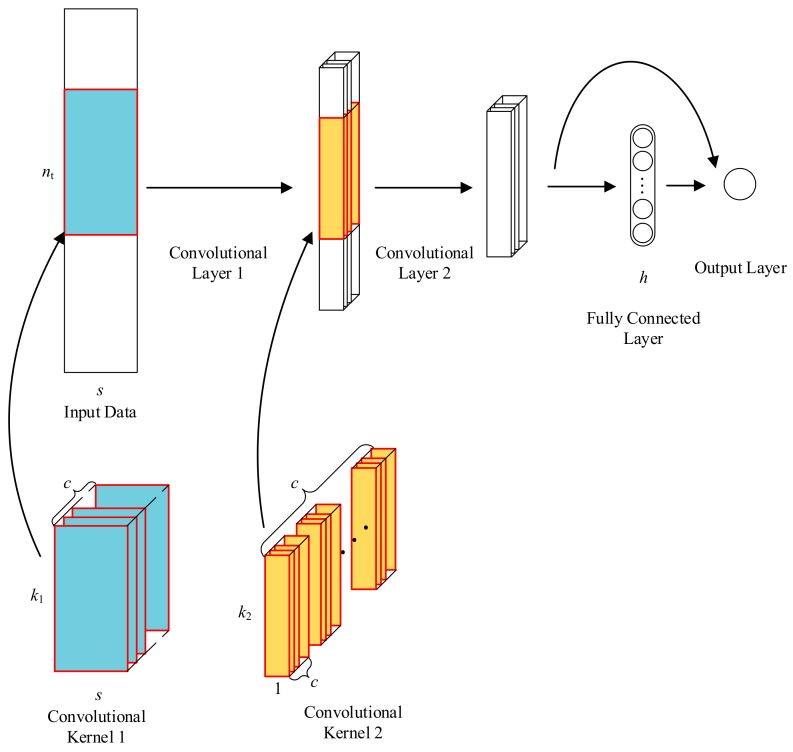
Architecture of the proposed response prediction model using convolutional neural networks.

The proposed CNN architecture is shown in Figure 2. There are in total four layers in the network architecture, two convolutional layers, one fully connected layer, and an output layer.

Inspired by Equation (8), to predict the non-reference response requires an exogenous input part and an auto-regressive part. Therefore, the architecture of the neural network is designed to contain two convolutional layers to process the time-domain vibration data. The first convolutional layer has kernels of size $k_{1}\times s$ to process input data in both dimensions. The kernels filter the input with a stride of 1. The number of convolutional kernels is set to c. Then, an element-wise nonlinear activation function is then executed on the convolutions. In this network, tanh function is used. The output of the first convolutional layer is a tensor of size $\left(n_{t}-k_{1}+1\right)\times 1\times c$.

The output of the first convolutional layer can be obtained by the following equation.

(12) $${\left[C_{1}\right]}_{j}=\tanh\left({\left[b_{1}\right]}_{j}+I*{\left[K_{1}\right]}_{j}\right),j=1,2,3,\cdots ,c$$

where $C_{1}$ is the output of the first convolutional layer, I is the input matrix of the virtual sensor, $K_{1}$ is the 2D kernel of the first convolutional layer, ${\left[b_{1}\right]}_{j}$ is the bias parameter.

In the second convolutional layer, to alleviate the difficulty in tuning the hyperparameters, the same number of kernels is used in this layer as in the first convolutional layer. Thus, in this layer, the output of the first convolutional layer is filtered with c kernels of size $k_{2}\times 1\times c$ with a stride of 1. Followed is also a tanh nonlinear activation function. Equation (13) shows the output of the second convolutional layer.

(13) $${\left[C_{2}\right]}_{j}=\tanh\left({\left[b_{2}\right]}_{j}+\sum_{i=1}^{c}{\left[K_{2}\right]}_{ij}*{\left[C_{1}\right]}_{i}\right),j=1,2,3,\cdots ,c$$

where $C_{2}$ is the output of the second convolutional layer, $K_{2}$ is a series of 3D kernels, and ${\left[b_{2}\right]}_{j}$ is a variable parameter.

The third layer is a fully connected layer. The number of neurons in this layer is h. The tanh function is used as the nonlinear activation function. V denotes the vector that reconstructed by the output of the second convolutional layer, H denotes the output of the hidden layer, $W_{h}$ and $b_{h}$ are the weight parameters and bias parameters in hidden layer respectively. The hidden layer can be shown as the following equation.

(14) $$H=\tanh\left(b_{h}+VW_{h}\right)$$

The final layer is the output layer. In neural networks, the output of different layers presents features of different depth. Theoretically, by combining the output in different layers, information in different depth can be contained and the results can be improved. Similar architecture can also be found in the network introduced by Jiang et al. [22], which is successfully utilized for time-series prediction. In the output layer. The output of the second convolutional layer and the fully connected layer are taken as the input of the output layer. No nonlinear activation function is used here. The output of the convolutional neural network can be obtained by

(15) $$O=VW_{o1}+HW_{o2}+b_{o}$$

where O is the output of the neural network, $W_{o1}$ and $W_{o2}$ are two weight parameters, and $b_{o}$ is the bias parameter.

## 4. Numerical Examples

To demonstrate the performance of the proposed method, two numerical examples are given in this response prediction section, i.e., a 6-degree-of-freedom (DOF) viscous damped mechanical system and a cantilever plate finite element model. In the field of structural dynamics, acceleration responses can be easily obtained using accelerometers in experimental and operational conditions, thus the accelerate responses are used here as the system input. Meanwhile, accelerate responses are widely utilized in industrial engineering for structural health monitoring, system identification, etc. Consequently, we choose acceleration responses as the output of the soft sensor.

The proposed virtual sensor model is established and implemented upon TensorFlow framework. The convolutional neural networks are constructed using the tf.nn.conv2d function. Before training the model, all variables of the neural network are initialized using the tf.truncated_normal function to generate values follow a normal distribution with 0 mean and standard deviation of 0.1. Considering the complexity and training steps of the neural network model for cantilever plate finite element model, a Graphics Processing Unit (GPU) is used to accelerate the model learning procedure.

### 4.1. 6-DOF Mechanical System

#### 4.1.1. Description of the 6-DOF System

**Figure 3:**
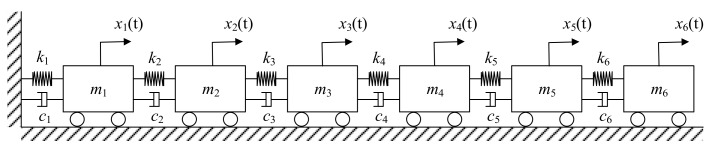
Schematic diagram of a 6-DOF mechanical system.

The 6-DOF model is shown in Figure 3, and the corresponding parameters are given in Table 1.

The dynamic equation of the 6-DOF model is described as

(16) $$M\ddot{x}\left(t\right)+C\dot{x}\left(t\right)+Kx\left(t\right)=F\left(t\right)$$

where $\ddot{x}\left(t\right)$, $\dot{x}\left(t\right)$ and x(t) are acceleration, velocity and displacement responses respectively. F(t) is excitation vector of the system. M, C and K are mass, damping and stiffness matrices respectively, and are given as:

(17) $$M=diag\left(m_{1},m_{2},m_{3},m_{4},m_{5},m_{6}\right),$$

(18) $$C=\left[\begin{array}{cccccc} c_{1}+c_{2} & -c_{2} & 0 & 0 & 0 & 0\\ -c_{2} & c_{2}+c_{3} & -c_{3} & 0 & 0 & 0\\ 0 & -c_{3} & c_{3}+c_{4} & -c_{4} & 0 & 0\\ 0 & 0 & -c_{4} & c_{4}+c_{5} & -c_{5} & 0\\ 0 & 0 & 0 & -c_{5} & c_{5}+c_{6} & -c_{6}\\ 0 & 0 & 0 & 0 & -c_{6} & c_{6} \end{array}\right],$$

(19) $$K=\left[\begin{array}{cccccc} k_{1}+k_{2} & -k_{2} & 0 & 0 & 0 & 0\\ -k_{2} & k_{2}+k_{3} & -k_{3} & 0 & 0 & 0\\ 0 & -k_{3} & k_{3}+k_{4} & -k_{4} & 0 & 0\\ 0 & 0 & -k_{4} & k_{4}+k_{5} & -k_{5} & 0\\ 0 & 0 & 0 & -k_{5} & k_{5}+k_{6} & -k_{6}\\ 0 & 0 & 0 & 0 & -k_{6} & k_{6} \end{array}\right]$$

#### 4.1.2. Virtual Sensor Model Description and Data Preparation

In this example, we use the acceleration response of the 1st, 3rd and 5th degree of freedom to predict the acceleration response of the 4th degree of freedom. The hyperparameters of the model used for the 6-DOF system are given in Table 2.

With label ${\ddot{x}}_{4}\left(t\right)$, the format of the input is a matrix I. According to the hyperparameters of the model, the size of the input matrix is 15 × 3. Thus, I is presented as

(20) $$I=\left[\begin{array}{ccc} {\ddot{x}}_{1}\left(t-1\right) & {\ddot{x}}_{3}\left(t-1\right) & {\ddot{x}}_{5}\left(t-1\right)\\ {\ddot{x}}_{1}\left(t-2\right) & {\ddot{x}}_{3}\left(t-2\right) & {\ddot{x}}_{5}\left(t-2\right)\\ {\ddot{x}}_{1}\left(t-3\right) & {\ddot{x}}_{3}\left(t-3\right) & {\ddot{x}}_{5}\left(t-3\right)\\ \vdots & \vdots & \vdots \\ {\ddot{x}}_{1}\left(t-15\right) & {\ddot{x}}_{3}\left(t-15\right) & {\ddot{x}}_{5}\left(t-15\right) \end{array}\right]$$

The data for training, validating, and testing the virtual sensor model are obtained by numerical stimulation in various load cases. The acceleration response series of all load cases are obtained by Newmark-*β* method, with Δt=0.001s, β=0.5, γ=0.25. The simulation time for each load case is 20 s. To alleviate the effect of the numerical noise, all signals are filtered using a 0–100 Hz low-pass filter.

To obtain the training dataset and validating dataset, the system is first excited by independent 80 dBw Gaussian white noise signals on each degree of freedom respectively to acquire 6 different response series. A superposition of the 6 signals is created as the training load case, since in a given linear system, responses from different load cases are able to be superposed to contain the structural properties in all 6 load cases.

To obtain a better performance in training, response series are first normalized. In structural dynamics, the positive and negative numbers indicate different directions of the acceleration. Thus, the normalization is performed by dividing the original response series with the maximum absolute value of the response in the whole dataset. In this model, the response series of 1st, 3rd, 4th and 5th degree of freedom are extracted from the original dataset, and the maximum value among all 4 response series is found. These response series are divided by the maximum value. Then, the time series response is reconstructed according to Equation (20).

There are total 20,000 time-series observations in the response series. The virtual sensor model uses responses from several discrete time steps before as the input to predict the following response. Thus, the response series cannot create 20,000 pairs of data for the neural network. Besides the size of the input data needs tuning. Hence, the dataset cannot contain response data in all 200,000 steps. Hence, here we state that the training dataset consists of the first 15,000 pairs of input data and labels. Then, the following 4000 pairs of data are used to validate the performance of the convolutional network in the training load case. Response series for producing testing datasets are generated using 6 more different 80 dBw Gaussian white noise series to excite the given 6-DOF system. Load Cases 1-1 to 1-6 represent the system excited by Gaussian white noise on different degrees of freedom respectively, as shown in Table 3. These response series are divided by the maximum value acquired in the normalization of the training data and then reconstructed using the same method of the training dataset. To evaluate the performance of the neural network model, we state that for each load case, the 19,000 pairs of input and output from step 1 to step 19,000 are selected as the testing datasets.

#### 4.1.3. Training and Evaluating the Model

The training process is performed using the build in Adam Optimizer with the mini-batch size of 100 and learning rate of 0.001. Training terminates after 10^5^ steps. The goal of the training process is to minimize the mean square error (MSE) of each mini-batch, which is shown in Equation (21).

(21) $$\mathrm{MSE}=\frac{1}{n}\sum_{i=1}^{n}{\left({\mathrm{observed}}_{i}-{\mathrm{predicted}}_{i}\right)}^{2}$$

**Figure 4:**
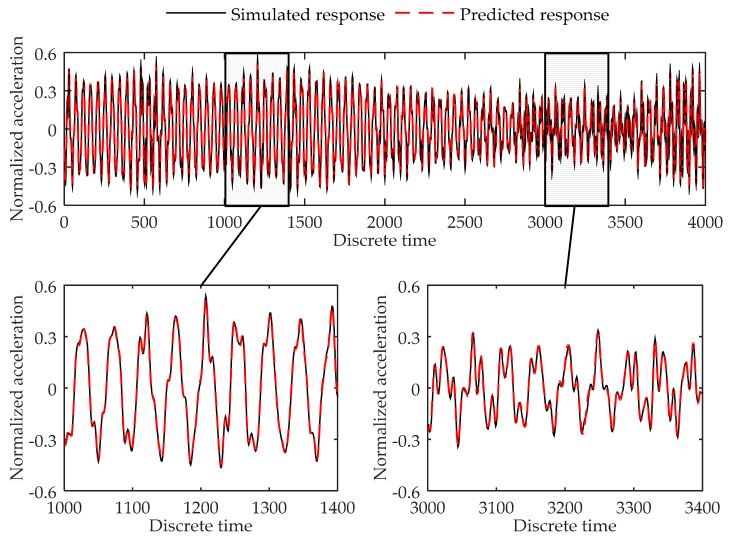
Comparison between the predicted response and simulated response of the 4th degree of freedom at the 6-DOF system in validation dataset.

Training error converges through the training process. At last, the training error in each training step oscillates around 1020 × 10^−4^, and the final training error is 1.17 × 10^−4^. The validation error is 1.32 × 10^−4^. Figure 4 shows the comparison of the simulated data and predicted output in validation dataset. As can be seen in the figure, the virtual sensor model is trained to be able to successfully predict the response of the 6-DOF system in the load case used for training the system.

**Figure 5:**
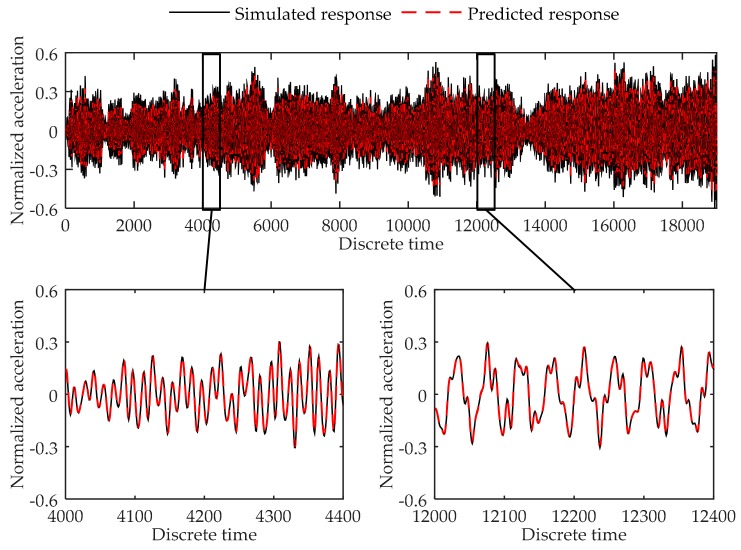
Comparison between the predicted response and true responses of the 4th degree of freedom at the 6-DOF system in Load Case 1-2.

**Figure 6:**
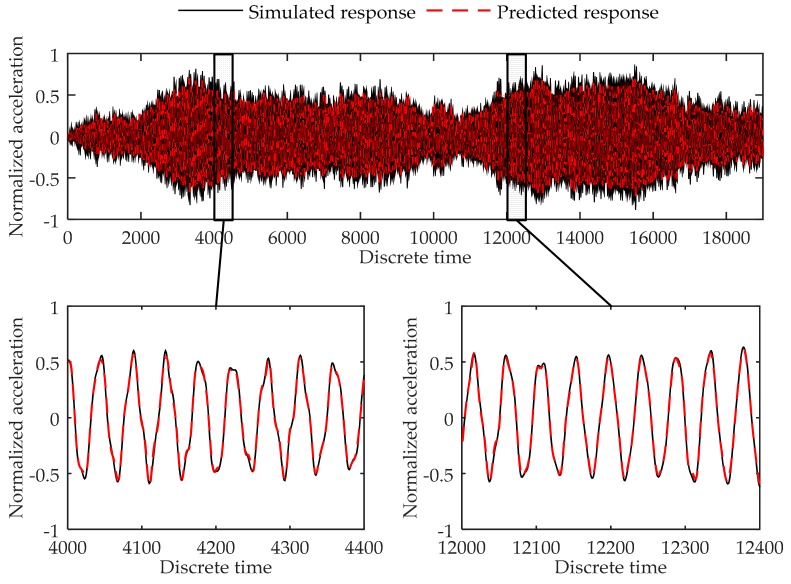
Comparison between the predicted response and true responses of the 4th degree of freedom at the 6-DOF system in Load Case 1-5.

The testing errors of the 19,000 data points are shown in Table 4. Figure 5 shows the simulated data and predicted response for Load Case 1-2, and Figure 6 shows the comparison of data in Load Case 1–5 as examples of the testing load cases. As can be seen in Table 4, the testing errors are similar to the training error and validation error, and the largest MSE error is 4.25 × 10^−4^, indicating the trained virtual sensor model is capable of accurately predicting the vibration responses. As shown in Figure 5 and Figure 6, the plots of the predicted responses and the simulated responses nearly coincide. Thus, in accordance with the testing errors and the plot of the results, it can be concluded that the proposed method is further demonstrated to be able to predict the structural response with high accuracy.

### 4.2. Cantilever Plate Finite Element Model

#### 4.2.1. Description of the Cantilever Plate Finite Element Model

**Figure 7:**
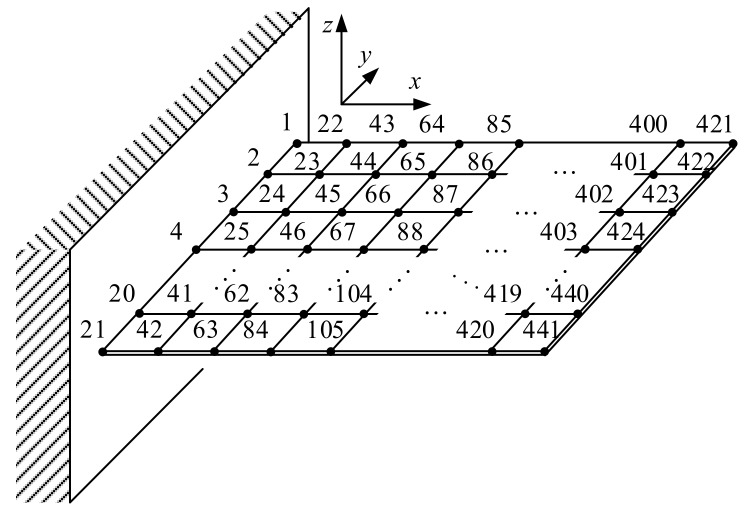
Schematic diagram of the cantilever plate finite element model.

Another numerical example, a finite element model of a cantilever plate, is used to test the performance of the proposed method. As shown in Figure 7, the cantilever plate is a square plate with side length of 1000 mm and thickness of 5 mm. The properties of the material are as follow: Young’s modulus is 72 GPa, Poisson’s ratio is 0.3, density is 2.69 × 10^3^ kg/m^3^. The plate is divided into 400 shell elements, and 441 nodes with labels from 1 to 441 are obtained. The labels of the nodes are shown in Figure 7. Excitations are loaded along the z-axis, and only the responses along z-axis are considered.

#### 4.2.2. Virtual Sensor Model Description and Data Preparation

Still, three nodes are considered as reference points. Responses for node 117, 253 and 421 are used to predict the response for node 352. The hyperparameters of the model are given in Table 5.

In the cantilever plate finite element model, we choose 20 nodes to excite on to create the response data of the training. The system is excited by independent 1 dBw Gaussian white noise on each of the chosen node respectively to acquire 20 s time series responses. The number of the nodes are 127, 132, 137, 142, 147, 232, 237, 242, 247, 252, 337, 342, 347, 352, 357, 421, 426, 431, 436 and 440. The testing load case is the superposition of all the time series responses obtained before.

Three kinds of load cases were used for testing. The first kind of load cases are the load cases that excitations loaded on the nodes included in the training load case. Four nodes are selected as examples of the 20 chosen nodes in the training load case, as Load Cases 2-1 to 2-4 in Table 6. The second kind of load cases is load cases in which the system is excited on the unchosen nodes. Another four nodes are selected as examples of the unchosen nodes as Load Cases 2-5 to 2-8. Finally, the third kind of load cases is excited on multiple nodes of the structure. In Load Case 2-9 and 2-10, 3 and 5 nodes are excited to acquire the response of the structure respectively. The numbers of nodes excited are listed in Table 6. In each load case, the excitation is 1 dBw independent Gaussian white noise series.

The acceleration response series of all load cases are obtained by Newmark-*β* method, with Δt=0.001s, β=0.5, γ=0.25. The simulation time for each load case is 20 s. To avoid numerical noise, the signals are filtered using a 0–100 Hz low-pass filter. The data is normalized and reconstructed with the same method in Section 4.1.2.

Training dataset is the former 15,000 data of all data in the training load case. Then the following 4000 data are used for the validation dataset. For each testing load case, response prediction was performed to predict the 19,000-time-step output from step 1 to step 19,000 in the response series. These data were used as testing datasets.

#### 4.2.3. Training and Evaluating the Model

**Figure 8:**
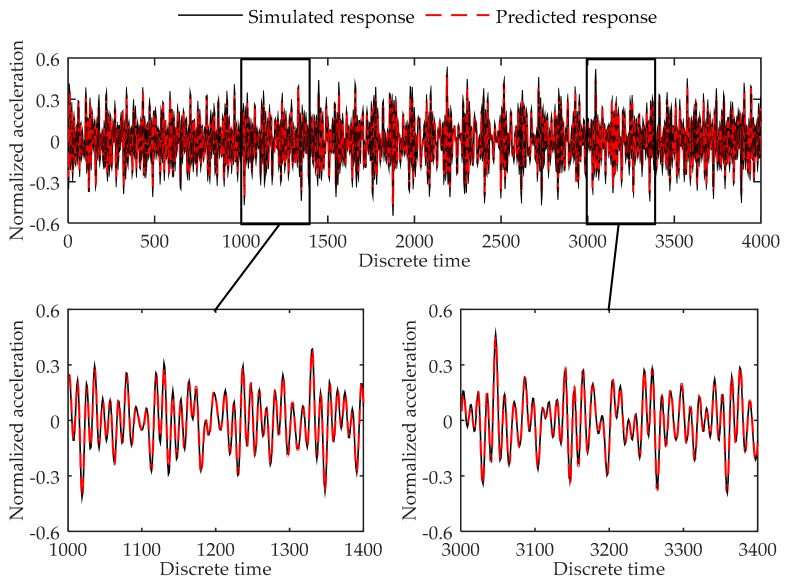
Comparison between the predicted response and true responses of node 352 at the cantilever plate finite element model in validation dataset.

In the training process, the build in Adam Optimizer is used to reduce the MSE of the mini-batch in each training step. The learning rate is set to 0.001, and the mini-batch size is 100. Training process stops after 500,000 training steps. The final training error is 5.47 × 10^−5^. Validation error is 7.76 × 10^−5^. The plot of the predicted result is shown in Figure 8. As can be seen, the trained model can accurately predict the response of the chosen location at the plate finite element model.

**Figure 9:**
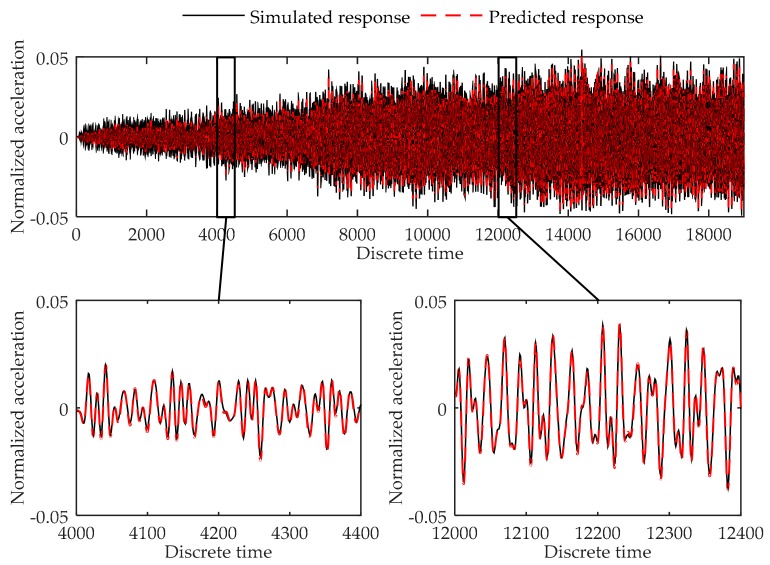
Comparison between the predicted response and simulated response of node 352 at the cantilever plate finite element model in Load Case 2-2.

**Figure 10:**
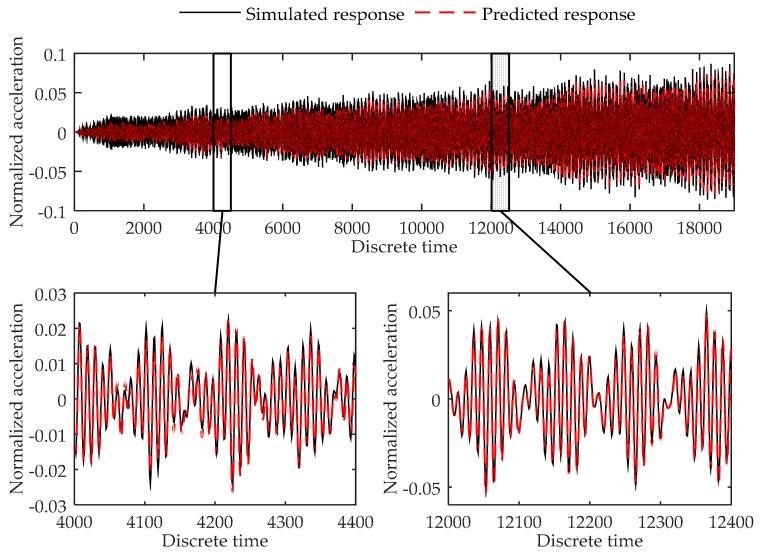
Comparison between the predicted response and simulated response of node 352 at the cantilever plate finite element model in Load Case 2-8.

**Figure 11:**
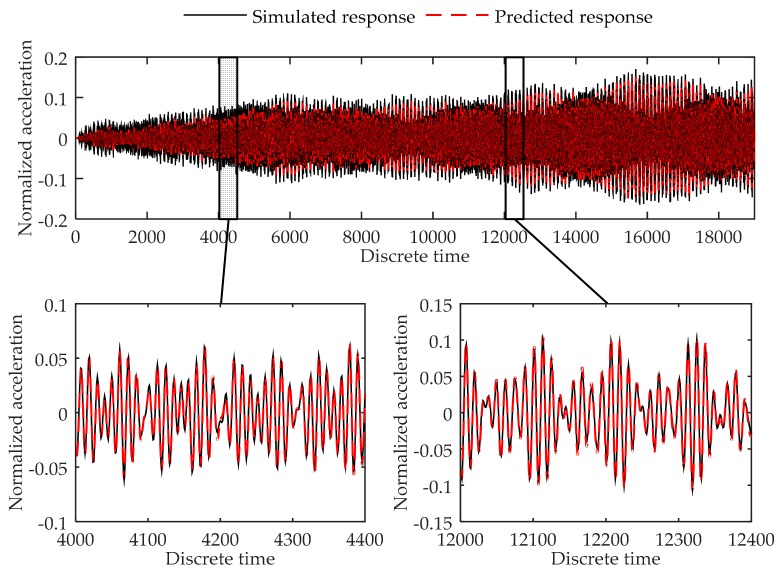
Comparison between the predicted response and simulated response of node 352 at the cantilever plate finite element model in Load Case 2-10.

Testing errors of Load Case 2-1 to 2-8 are given in Table 7. Figure 9, Figure 10 and Figure 11 show the plots of the testing load cases under Load Case 2-2, Load Case 2-8, and Load Case 2-10 respectively as the examples of different kinds of load cases. Testing results indicate that the model acquired high accuracy in all test load cases. As can be concluded, the proposed method can successfully predict the response of the cantilever plate finite element model in both training load case and testing load cases.

## 5. Experimental Example

In this section, a simply supported beam experiment is conducted to show the good performance of the proposed model in real structural systems. Then, the model is compared with a modal-model-based virtual sensor method to further demonstrate the excellence of the novel virtual sensor model. The modal-model-based virtual sensor uses response from other physical sensors to estimate the modal parameters then calculate the desired response together with the mode shapes of the structure. The construction of the virtual sensor model and the preparation of the datasets are the same in Section 4. In Section 5.1, the experiment system is introduced. Section 5.2 explains the virtual sensor model and the data preparation procedure. Section 5.3 presents the experimental results and the comparison with the modal-model-based virtual sensor.

### 5.1. Description of the Experiment System

**Figure 12:**
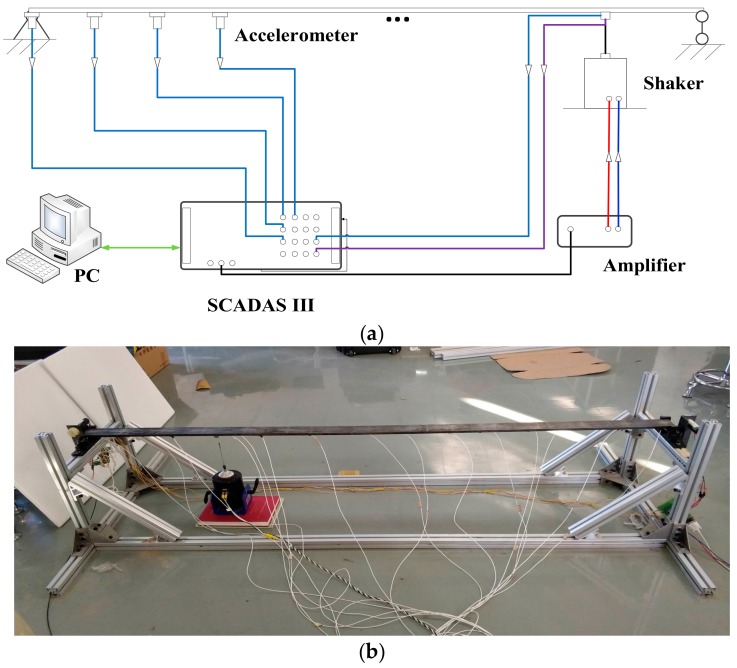
(**a**) Diagrams of experiment system; (**b**) Photo of the experiment system.

**Figure 13:**
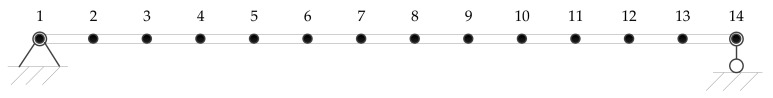
Locations on the experimental beam for the distribution of sensors and the position shaker.

The simply supported beam experimental system is derived and simplified from a time-varying experimental system. For more information please refer to [23]. The experiment system is established as seen in Figure 12a. The experimental structure is a simply supported steel beam, the parameters of which are listed in Table 8. A picture of the experimental structure is given in Figure 12b. The beam is evenly divided into 13 parts, and 14 locations are obtained on the beam to distribute sensors and position the shaker as shown in Figure 13.

To measure the acceleration responses of the beam, fourteen piezoelectric accelerometers (sensitivity: E100 mV/g) are uniformly distributed along the axial direction of the beam. For acquiring experiment data under different load cases, the position of the shaker changes from location 2 to location 13. The acceleration response signals and the force signal of the shaker are acquired using an LMS SCADAS III system, the response data are then recorded in the directly linked computer with the assistant of LMS TestLab software.

### 5.2. Virtual Sensor Model and Data Preparation

In this model, we chose six locations as reference points. Measured responses of location 3, 5, 7, 9, 11 and 13 are used to predict the response of location 6. The hyperparameters of the model are given in Table 9.

The excitations are loaded on location 3, 5, 7, 9 and 11 respectively to acquire five time-series response signals for training the virtual sensor. The system is excited by the shaker, connected to an amplifier, controlled by the PC using the TestLab software. The excitations are independent Gaussian white noise with a bandwidth of 0–256 Hz.

Testing load cases can be regarded as two kinds. The first kind of load cases are the same as those used for training, but the excitations series are different and independent. In the second kind of load cases, excitations are loaded on the unchosen locations. Table 10 shows the number of locations excited in different testing load cases. Among which, Load Cases 3-1 to 3-5 are the first kind of load cases, and Load Cases 3-6 to 3-12 are the second kind of load cases.

The signals are sampled with a sampling frequency of 2048 Hz, the record length is 10 s. Then, signals are filtered using a 0–256 Hz low-pass filter. The data is normalized and reconstructed with the same method in Section 4.1.2.

The beginning 15,000 data from each training load case are obtained and combined together as a training dataset of 75,000 pairs of data. Since the training dataset is not the superposition of the signals from different load cases, the validation datasets are the responses at the following 4000 steps in each training load case. For each testing load case, response prediction was performed to predict the 19,000-time-step output from step 1 to step 19,000 in the response series, similar to the former two numerical examples. These data were used as testing datasets.

### 5.3. Experiment Results

Same strategy as the numerical examples in the training process of the virtual sensor model is used. The build-in Adam Optimizer is used, and the goal of the training process is to minimize the MSE of the mini-batch. The learning rate is set to 0.0001, and the mini-batch size is 150. Training process stops after 2,000,000 training steps. The training errors and validation errors are listed in Table 11.

To further demonstrate the performance of the virtual sensor model using convolutional neural networks, it is compared to a modal-model-based virtual sensor using ordinary least square solution [6]. The modal-model-based sensor used here requires natural mode shape vectors of the structure. They can be acquired either by the analysis of a finite element model, or by the estimation of the experiment system. In this paper, we use LMS Testlab software to calculate the mode shapes. Since the modal-model-based method requires the number of mode shapes should be no larger than the number of input sensors, here we use six lowest modes.

The reason for not using the data-driven virtual sensors introduced in [7] is that the introduced minimum mean square error estimation is capable of detecting and identifying the faulty sensors, but is not able to reconstruct the faulty sensor with high accuracy as mentioned in the literature. Furthermore, spatiotemporal correlation, which is used to accurately estimate the responses in the paper, is stationary, cannot be used in real-time estimation.

**Figure 14:**
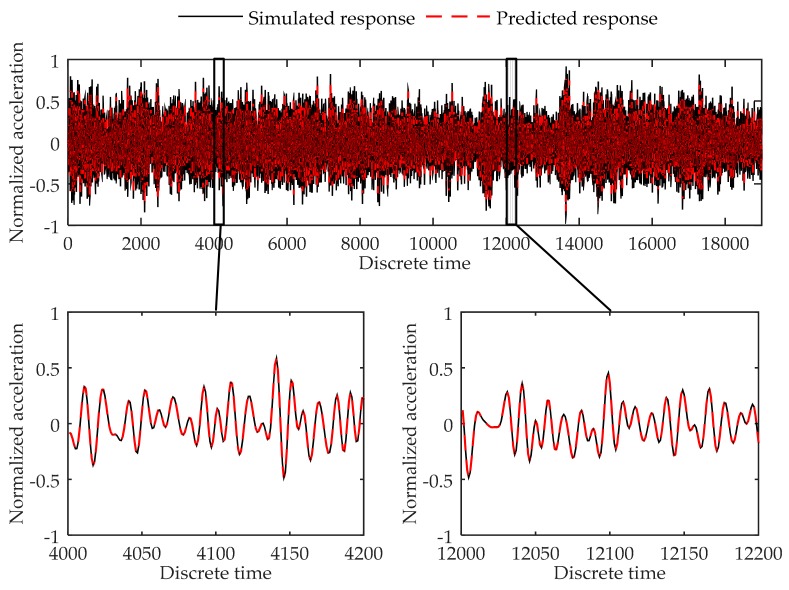
Comparison between the predicted response and real response of the location 6 at the simply supported beam experiment in Load Case 3-2.

**Figure 15:**
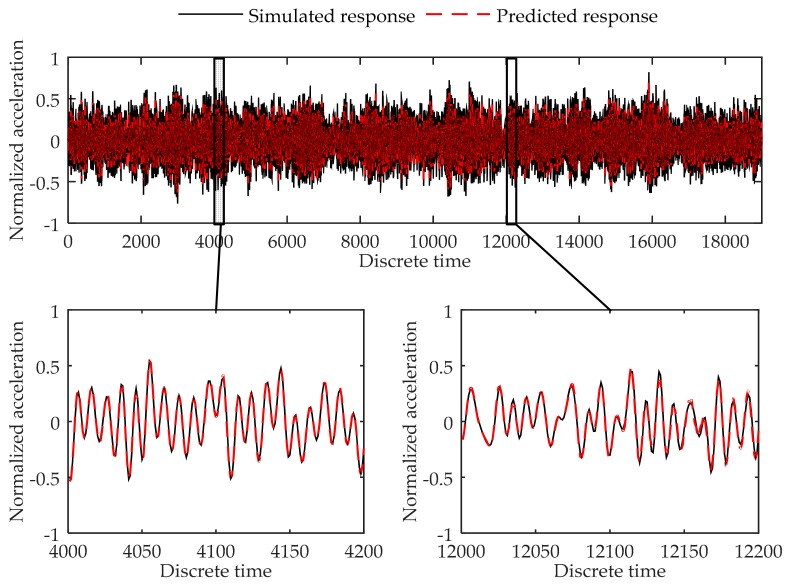
Comparison between the predicted response and real response of the location 6 at the simply supported beam experiment in Load Case 3-8.

Testing errors of Load Cases 3-1 to 3-12 for both virtual sensors using convolutional neural network and the modal-model-based virtual sensor are given in Table 12. The plots of the results in two examples of different kinds of load cases, Load Cases 3-2 and 3-8, are shown in Figure 14 and Figure 15, respectively. Testing results show high accuracy in most load cases.

**Figure 16:**
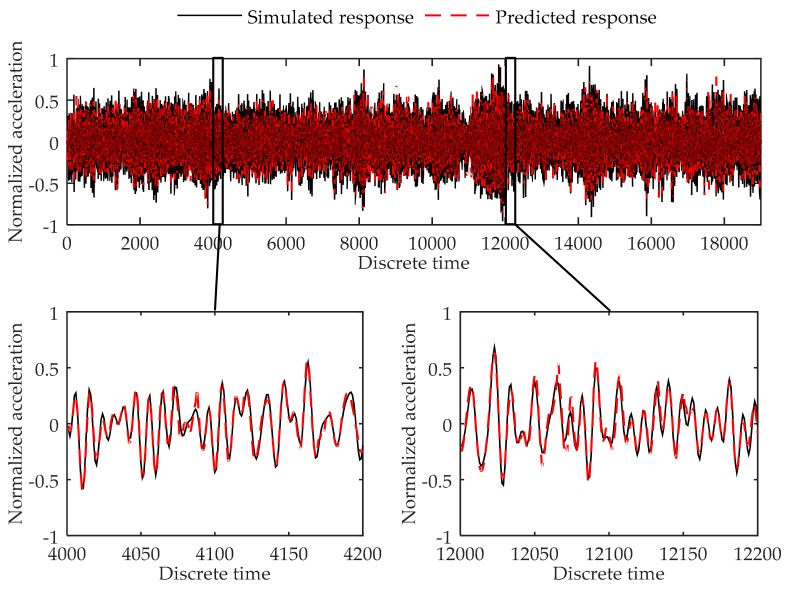
Comparison between the predicted response and real response of the location 6 at the simply supported beam experiment in Load Case 3-10.

As can be seen from Table 12, testing errors in the load cases that are not included in the training load cases are relatively large. However, the novel virtual sensor model still contains similar accuracy as the modal-model-based virtual sensor. In Figure 16, the plots of real responses and predicted responses are given. It can be clearly seen that the proposed model is still able to predict the vibration responses, even the testing errors are not as good as in the load cases included in the training procedure. Thus, it can be concluded that the proposed method can successfully predict the response of the experiment system with a high accuracy.

In addition, in the modal-model-based virtual sensor, the more input sensors available, the more accurate the estimation can be. This is because as the number of input sensor increases, not only the accuracy of the ordinary least square increases, but the number of available active natural mode shapes increases. Thus, another advantage of using the novel virtual sensor using convolutional neural networks is dealing with situations when physics sensors are limited.

## 6. Discussions

In this section, the choosing of activation function and the selection of hyperparameters are briefly discussed. In Section 6.1, different activation functions are discussed. In Section 6.2, a brief advice in selecting the hyperparameters is given.

### 6.1. Different Activation Functions

In the area of deep learning and neural network, three activation functions are now widely used in building deep neural networks. They are tanh function, sigmoid function and Rectified Linear Unit (ReLU) function, shown in Equations (11), (22) and (23), respectively:

(22) $$sigmoid\left(x\right)={\left(1+e^{-x}\right)}^{-1}$$

(23) ReLU(x)=max(0,x)

To justify the performance of different activation functions, different activation functions are used in the experiment example described in Section 5. For each activation function, the virtual sensor model is trained and evaluated five times, and the average training errors and average validation errors are listed in Table 13.

As can be concluded from Table 13, validation error of the model using tanh function obtained a higher accuracy than the sigmoid function. Furthermore, the validation errors of virtual sensor model using tanh function are similar to that of the model using ReLU. Here we simply selected tanh function because it has been widely used in neural networks for solving regression problems in accordance with the authors’ knowledge.

### 6.2. Hyperparameters of the Virtual Sensor Model

Like any neural network, the tuning of the hyperparameters is essential and still a hot issue in the research of artificial neural networks (ANNs). In ANNs, the features are automatically learned and then used for different tasks, making them require no manually input engineering features. However, to achieve the best result, the hyperparameters need to be tuned. Hyperparameters are basically divided into two groups to define the learning process (e.g., learning rate or mini-batch size) and the architecture (e.g., the numbers of the layers) respectively. Although there are researches working on methods to automatically select hyperparameters through optimization or other algorithms, researchers still mainly tuning hyperparameters manually.

The hyperparameters used for defining the architecture affects the performance of the neural network by deciding the number of parameters used in the model. If the architecture of the virtual sensor model is too small, which means the model contains only a few parameters, the model is not able to predict an accurate response. Usually, as the number of parameters increases, the accuracy of the predicting results improves, but the computational cost increases. However, if the architecture of the model is too large, indicating the model has too many parameters, the overfitting problem will arise. The overfitted model has a quite high accuracy in training dataset, but it cannot contain the same accuracy in testing datasets, which indicates the generalization ability of the model decreases. Thus, in the tuning process of the hyperparameters, we focus on obtaining a high training accuracy and validation accuracy of the model. Meanwhile, the training error and validation error should be close to each other.

In this paper, there are six different hyperparameters used for defining the architecture of the convolutional neural network. To give proper advice in selecting the hyperparameters, several rounds of training and testing were performed using different hyperparameters. The number of reference sensors, s, is decided according to the circumstances, usually, an integer larger than 2 is recommended. The size of the convolutional kernels are denoted $k_{1}$ and $k_{2}$. The size of the first kernel, $k_{1}$, is recommended to be around 8 to 20 according to the complexity of the structure. Similarly, the recommended value for $k_{2}$ is 5 to 10. The total number of discrete response in input data, $n_{t}$, should be a little larger than the sum of $k_{1}$ and $k_{2}$, usually around 15 to 40 according to the complexity of the structure. The number of the output channel, c, indicates the number of features that learned by the convolutional neural network. The value of c recommends to be around 20 to 40. If the structure is quite complex, the value can be increased, but better not larger than 50. The number of neurons in the fully connected layer is denoted as h. The value is recommended as an integer around 5 to 10.

## 7. Conclusions

This paper proposes a data-driven response virtual sensor with partial vibration measurements using convolutional neural network. We use transmissibility functions as prior knowledge for building the virtual sensor model. Then, a four-layer neural network, consists of two convolutional layers, one fully connected layer, and one output layer, is introduced. We verify the performance of the novel virtual sensor model with two numerical examples and one experimental example. The virtual sensor using convolutional neural network outperforms a modal-model-based virtual sensor in the experimental example. This further illustrates the excellence of the proposed method.

The proposed data-driven virtual sensor requires only sufficient data for training the neural network. However, in model-based methods, a finite element model is usually required, the establishment of which is rather time-consuming and effort taking. Compared to the modal-model-based virtual sensor, the proposed method can obtain better accuracy. Furthermore, since the transmissibility functions are used as the prior knowledge, the proposed method only requires a small number of input sensors to obtain high accuracy, while in the modal-model-based method, available sensors need to be redundant to ensure adequate active mode shapes are selected.

The high accuracy of the method makes it possible to implement it in real structural systems to generate sufficient vibration data under working conditions when available physics sensors are limited. These data can further help monitoring health condition and detecting structural damages.

## Figures and Tables

**Table 1:** Parameters of the 6-DOF system.

Parameters	Values
Mass/kg	*m*_1_ = 0.2, *m*_2_ = 0.1, *m*_3_ = 0.3, *m*_4_ = 0.5, *m*_5_ = 0.2, *m*_6_ = 0.3
Damping/N·m^−1^·s	*c*_1_ = *c*_2_ = *c*_3_ = *c*_4_ = *c*_5_ = *c*_6_ = 2.5
Stiffness/N·m^−1^	*k*_1_ = *k*_2_ = *k*_3_ = *k*_4_ = *k*_5_ = *k*_6_ = 1 × 10^5^

**Table 2:** Hyperparameters of the virtual sensor model for response prediction of the 6-DOF system.

Hyperparameters	Values	Hyperparameters	Values
$n_{t}$	15	$k_{2}$	5
s	3	c	10
$k_{1}$	6	h	5

**Table 3:** Number of degree of freedom (DOF) excited in Load Cases 1-1 to 1-6.

Load Cases	No. of DOF	Load Cases	No. of DOF
Load Case 1-1	1	Load Case 1-4	4
Load Case 1-2	2	Load Case 1-5	5
Load Case 1-3	3	Load Case 1-6	6

**Table 4:** Testing error of Load Cases 1-1 to 1-6.

Load Cases	MSE	Load Cases	MSE
Load Case 1-1	2.75 × 10^−5^	Load Case 1-4	1.03 × 10^−4^
Load Case 1-2	2.00 × 10^−5^	Load Case 1-5	4.25 × 10^−4^
Load Case 1-3	3.48 × 10^−5^	Load Case 1-6	1.10 × 10^−4^

**Table 5:** Hyperparameters of the virtual sensor model for response prediction of the cantilever plate finite element model.

Hyperparameters	Values	Hyperparameters	Values
$n_{t}$	24	$k_{2}$	5
s	3	c	20
$k_{1}$	8	h	5

**Table 6:** Number of nodes excited in Load Case 2-1 to 2-8.

Load Cases	No. of Nodes	Load Cases	No. of Nodes
Load Case 2-1	132	Load Case 2-6	185
Load Case 2-2	247	Load Case 2-7	285
Load Case 2-3	337	Load Case 2-8	385
Load Case 2-4	426	Load Case 2-9	185, 274, 350
Load Case 2-5	85	Load Case 2-10	33, 93, 153, 213, 373

**Table 7:** Testing errors (MSE) of Load Case 2-1 to 2-8.

Load Cases	Testing Errors	Load Cases	Testing Errors
Load Case 2-1	3.70 × 10^−6^	Load Case 2-6	7.85 × 10^−7^
Load Case 2-2	1.50 × 10^−6^	Load Case 2-7	3.07 × 10^−6^
Load Case 2-3	4.75 × 10^−6^	Load Case 2-8	3.78 × 10^−6^
Load Case 2-4	1.41 × 10^−5^	Load Case 2-9	7.70 × 10^−6^
Load Case 2-5	2.82 × 10^−6^	Load Case 2-10	1.08 × 10^−5^

**Table 8:** Basic parameters of the simply supported beam.

Length/mm	Width/mm	Height/mm	Weight/kg
2000	60	10	9.420

**Table 9:** Hyperparameters of the virtual sensor model for response prediction of simply supported beam experiment.

Hyperparameters	Values	Hyperparameters	Values
$n_{t}$	40	$k_{2}$	8
s	6	c	40
$k_{1}$	20	h	10

**Table 10:** Number of locations excited in Load Case 3-1 to 3-12.

Load Cases	No. of Locations	Load Cases	No. of Locations
Load Case 3-1	3	Load Case 3-7	4
Load Case 3-2	5	Load Case 3-8	6
Load Case 3-3	7	Load Case 3-9	8
Load Case 3-4	9	Load Case 3-10	10
Load Case 3-5	11	Load Case 3-11	12
Load Case 3-6	2	Load Case 3-12	13

**Table 11:** Training errors (MSE) and validation errors (MSE) in all training load cases.

Excited Locations	Training Errors	Validation Errors
Location 3	6.42 × 10^−6^	4.67 × 10^−6^
Location 5	4.64 × 10^−6^	4.46 × 10^−6^
Location 7	7.63 × 10^−6^	7.14 × 10^−6^
Location 9	5.85 × 10^−6^	7.45 × 10^−6^
Location 11	6.21 × 10^−6^	4.84 × 10^−6^

**Table 12:** Testing errors (MSE) of Load Case 3-1 to 3-12.

Load Cases	Virtual Sensor Using CNN	Modal-Model-Based Virtual Sensor
Load Case 3-1	1.18 × 10^−5^	1.24 × 10^−4^
Load Case 3-2	7.92 × 10^−6^	3.28 × 10^−4^
Load Case 3-3	1.88 × 10^−5^	3.59 × 10^−3^
Load Case 3-4	1.51 × 10^−5^	4.83 × 10^−3^
Load Case 3-5	7.43 × 10^−6^	7.85 × 10^−3^
Load Case 3-6	4.76 × 10^−4^	2.17 × 10^−3^
Load Case 3-7	7.48 × 10^−4^	3.35 × 10^−3^
Load Case 3-8	3.90 × 10^−4^	3.97 × 10^−3^
Load Case 3-9	2.21 × 10^−3^	5.18 × 10^−3^
Load Case 3-10	4.18 × 10^−3^	2.68 × 10^−3^
Load Case 3-11	4.46 × 10^−3^	6.55 × 10^−4^
Load Case 3-12	1.40 × 10^−3^	3.83 × 10^−3^

**Table 13:** Average validation errors (MSE) for virtual sensor using convolutional neural network in experiment example under different training load cases with different activation functions.

Excited Locations	Tanh Function	Sigmoid Function	ReLU
Location 3	5.56× 10^−6^	3.24 × 10^−5^	4.62 × 10^−6^
Location 5	4.37 × 10^−6^	3.25 × 10^−5^	8.93 × 10^−6^
Location 7	7.70 × 10^−6^	6.32 × 10^−5^	5.31 × 10^−6^
Location 9	7.84 × 10^−6^	4.67 × 10^−5^	9.33 × 10^−6^
Location 11	5.23 × 10^−6^	3.65 × 10^−5^	4.61 × 10^−6^
